# BGP: identifying gene-specific branching dynamics from single-cell data with a branching Gaussian process

**DOI:** 10.1186/s13059-018-1440-2

**Published:** 2018-05-29

**Authors:** Alexis Boukouvalas, James Hensman, Magnus Rattray

**Affiliations:** 1 Division of Informatics, Imaging and Data Sciences, Faculty of Biology, Medicine and Health, University of Manchester, Oxford Road, Manchester, UK; 2 Prowler.io, Cambridge, UK

**Keywords:** Single cell RNA-seq, Gaussian process, Branching dynamics

## Abstract

High-throughput single-cell gene expression experiments can be used to uncover branching dynamics in cell populations undergoing differentiation through pseudotime methods. We develop the branching Gaussian process (BGP), a non-parametric model that is able to identify branching dynamics for individual genes and provide an estimate of branching times for each gene with an associated credible region. We demonstrate the effectiveness of our method on simulated data, a single-cell RNA-seq haematopoiesis study and mouse embryonic stem cells generated using droplet barcoding. The method is robust to high levels of technical variation and dropout, which are common in single-cell data.

**Electronic supplementary material:**

The online version of this article (10.1186/s13059-018-1440-2) contains supplementary material, which is available to authorized users.

## Background

Single-cell gene expression data can be used to uncover cellular progression through different states of a temporal transformation, e.g. during development, differentiation or disease. As single-cell protocols improve, a flurry of methods have been proposed to model branching of cellular trajectories to alternative cell fates [1–4]. In these and similar methods, pseudotime is estimated and a global branching structure is inferred. Our focus in this paper is on a downstream analysis method that can subsequently be used to model branching gene expression dynamics for individual genes. We are interested in discovering which genes follow the global cellular branching dynamics and whether these genes branch early or late with respect to the global cellular branching time. Recently, [3] proposed the branch expression analysis modelling (BEAM) approach, which uses penalised splines to infer the individual gene branching time. Here, we propose an alternative non-parametric method to model gene expression branching dynamics. We develop a probabilistic generative model of branching dynamics that can be used to assess the evidence for branching and to provide a posterior estimate of the branching time. The posterior distribution over branching time can be used to identify the most likely branching time for each gene as well as an associated credible region capturing our uncertainty in the estimate.

Our approach is based on Gaussian processes (GPs), which are a class of flexible non-parametric probabilistic models. GPs have a long history in temporal and spatial statistics and have gained popularity in many areas of machine learning, including multivariate regression, classification and dimensionality reduction [5]. GPs have been used for dimensionality reduction of single-cell expression data [6, 7] and more recently for pseudotime estimation where the effect of uncertainty in the inferred pseudotime can be quantified [8] and capture time can be included as prior information [9, 10]. GP-based methods have also been used for modelling global cellular branching dynamics from single-cell data after assigning pseudotime to cells [11].

Here, we build on the work of [12], who developed a GP model to identify when two gene expression time-course data sets first diverge from one another. They defined a novel GP covariance function that constrains two functions to intersect at a single point. The divergence time is inferred by numerically approximating the posterior using a simple histogram approach. The model identifies when a gene first becomes differentially expressed in time-course gene expression data under control and perturbed conditions. In their approach, all data points are labelled with the branch that generated them and the ordering of time points is assumed known. Although similar to the problem of modelling branching in single-cell data after pseudotime is inferred, this two-sample time-series method cannot be applied directly to our problem because we have to allow for uncertainty regarding which branch each cell belongs to.

Also closely related to the present work, the overlapping mixture of GPs (OMGP) [13] is a mixture model for time-series data where the mixture components are GP functions and the data at any time can be assigned to any of the components. For single-cell data, after pseudotime is assigned to each cell, then the OMGP model can be used to assign cells to different trajectories. The cell labels do not have to be known in advance and can be inferred through fitting the model to data. However, in OMGP models, the cellular trajectories are independent rather than branching. The OMGP model has been applied to single-cell data to infer global cell branching times [11] but as OMGP assumes the latent functions are independent without any branching, a heuristic based on a piecewise linear fit of the log likelihood surface is proposed to identify the most likely branching times. This is problematic, since OMGP does not provide a proper generative model of branching dynamics and therefore, it is not clear how to compute the posterior distribution over the branching time.

Our main methodological contribution here is to generalise the OMGP model to account explicitly for dependence between the functions in the mixture model. Specifically, we consider that the functions branch, as in [12]. This allowed us to develop a probabilistic model over branching cellular trajectories where the assignment of cells to branches is not known in advance. Our new branching GP (BGP) model allows us to calculate the posterior distribution over branching time for each gene while allowing for uncertainty in the branch labels for each cell. This uncertainty is especially important for early-branching genes, since cells are not labelled with a branch prior to the global cellular branching time, which we assume is known.

A naive implementation of GP models scales cubically with the size of the data. As increasing numbers of cells can be profiled in new single-cell protocols, we ensure the scalability of our approach by employing two complementary approaches. Firstly, we use sparse inference [14, 15], which allows model fitting to scale with the number of inducing points. The latter is a user-defined value that trades off model accuracy and training time. Specifically for *N* cells, naive covariance inversion scales as *O*(*N*^3^) while under sparse inference with *k* inducing points, it scales as *O*(*k*^2^*N*) where typically *k*≪*N*. Secondly, we provide an open-source implementation that leverages the GPflow library [16], which both simplifies the implementation due to automatic symbolic differentiation and allows for the necessary matrix operations to be computed in parallel across many CPU nodes or GPUs.

The paper is organised as follows. We first give an overview of the BGP model, including how inference is performed in a scalable manner. We discuss how uncertainty is quantified and represented in a full posterior distribution on the branching time for each gene. We contrast the performance of the BGP method to two recently published methods, the mixture of factor analysers (MFA) [17] and the BEAM approach [3], in a synthetic study across a variety of simulated scenarios. We apply the BGP model to data from a haematopoiesis study [18] and to single-cell mouse embryonic stem cell data generated using droplet barcoding [19]. We conclude with a summary of our findings and a discussion of possible future research directions.

## Results and discussion

### Overview of BGP

Before applying the BGP method, we require the pseudotime for each cell and the global branching pattern of the cells. In our experiments, we used results from the Monocle reversed graph embedding approach, termed DDRTree [3], and the diffusion pseudotime (DPT) [1] and the wishbone [2] approaches. However, BGP can be based on any method that estimates pseudotime and a gene-wide cell branching association. For a recent review, see [20].

OMGP [13] is a mixture model of independent GPs that is able to associate an observation with the generating GP. The authors term this the association problem and derive a variational inference algorithm for independent GPs. Our work extends the OMGP model in two directions: firstly, we remove the assumption of latent function independence and allow dependent GPs as required by a branching model. Secondly, we provide a sparse inducing point approximation that allows for scalable inference.

Let *F* be a BGP evaluated for *N* data points (cells) with *M* latent functions. *Z*∈{0,1}^*N*×*M*^ indicates which branch each cell comes from. The number of latent functions for a single branching point is *M*=3, as we have separate latent functions for the trunk and each branch. The likelihood is $p\left (Y|F,Z\right)={\mathcal N}\left (Y|ZF,\sigma ^{2}I\right)$ and as in [13], we place a categorical prior on the indicator matrix $p(Z) =\prod _{n=1}^{N}\prod _{m=1}^{M}\left [\Pi \right ]_{n,m}^{[Z]_{nm}}$. We place a GP prior on the latent functions $p\left (F | t_{b}\right) =\mathcal {GP}\left (0,K | t_{b}\right)$, which constrains the latent functions to branch at pseudotime *t*_*b*_. Note that the latter does not factorise as in [13], as the latent functions are dependent. Further details of the model derivation and inference scheme used are provided in ‘Methods’ and a detailed derivation, including the inducing point approximation, is provided in the supplementary material (Additional file 1: Section 2).

Global branching labels, such as those provided by DDRTree, can provide an informative prior *p*(*Z*) for all genes. The prior before the global branching point is uninformative, as no global assignment is available. This is relevant for early-branching genes, which may start branching earlier than the global cellular branching. After the global branching point, the prior favours an increased assignment probability to the globally assigned branch. If the prior places significant mass on the alternative assignment, the resulting assignment may differ from the global allocation given enough evidence from the likelihood term. This allows the model to correct mislabelled cells as well as account for sources of noise in the data, such as dropout for lowly expressed genes. However, in our single-cell case studies, we use a strong prior assignment probability of 0.99 to avoid cell reassignment after the global branching time. This simplifies the interpretation of the results, as cells do not switch their global branch assignment. Nevertheless, other constructions are possible with this model, e.g. the distance of the cells from the global branching time may be used to adjust the associated prior uncertainty.

The model hyperparameters are fitted by maximising a bound on the log-likelihood. The log-likelihood is not analytically tractable, as it involves integrating out the indicator matrix *Z* and therefore, we use a variational approximation. A lower bound is available using Jensen’s inequality:

1 $$ \log p\left(Y|F\right) \geq\mathbb{E}_{q(Z)}\left[\log p\left(Y|F,Z\right)\right]-KL\left[q(Z)||p(Z)\right],  $$

where we use a mean-field approximation *q*(*Z,F*)=*q*(*Z*)*q*(*F*) with the latent functions *F* independent of the association indicators *Z* and $q(Z) = \prod _{nm} \phi _{nm}$. The *ϕ*_*nm*_ approximates the posterior probability of cell *n* belonging to branch *m*. The latter is either the trunk state or one of the two branches for the single branching considered in the applications here. Then, *F* can be integrated out to get the marginal likelihood *p*(*Y*).

The branching time posterior probability is calculated using the approximate marginal likelihood evaluated at a set of candidate branching points *S*_*B*_ of size *N*_*b*_. The posterior for a candidate branching time *c* is

2 $$ p(t_{b}=c|Y) = \frac{p(Y|t_{b}=c)}{\sum_{i\in S_{B}} p(Y|t_{b}=i)}.  $$

The marginal likelihood of the model can also be used to calculate the Bayes factor of branching versus not branching (assuming equal priors). This is used to rank genes by how likely it is that their expression exhibits branching. By numerically integrating out the uncertainty of the branching location, the logged Bayes factor *r*_*g*_ includes the effect of posterior uncertainty in the branching location:

3 $${} \begin{aligned} r_{g} &= \log \frac{P(0<t_{b}<1|Y)}{P(t_{b} \rightarrow \infty)|Y)} \\ &= \log\left[\frac{1}{N_{b}} \sum_{i \in S_{B}} p\left(Y|t_{b} = i\right)\right] - \log\left[p\left(Y|t_{b} \rightarrow \infty)\right)\right], \end{aligned}  $$

where *t*_*b*_=*∞* specifies that the model does not branch and we have assumed equal prior probabilities for branching and not branching.

**Fig. 1:**
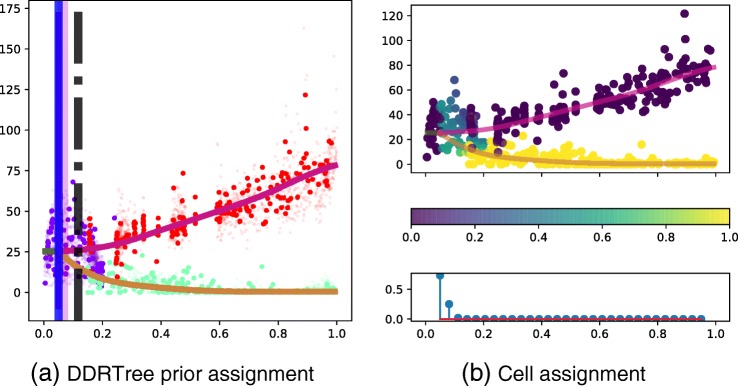
Haematopoiesis gene expression, showing the BGP fit for the MPO gene. **a** The Wishbone branching assignment is shown for each cell along with the global branching time (black dashed line), the most likely branching time (blue solid line) and posterior branching time uncertainty (magenta background). The sample of cells used to fit the BGP model is shown with larger markers. **b** The posterior cell assignment is shown in the top subpanel. In the bottom subpanel, the posterior branching time is shown. Pseudotime is shown on the horizontal axis of all plots. **a**, **b** Gene expression is depicted on the vertical axis. **c** The posterior branching probability BGP branching Gaussian process

An example of the BGP model fit is shown in Fig. 1b. The uncertainty in the cell branch association is shown in conjunction with the posterior on the branching times. For visualisation, the cell assignment to the top branch is shown. We see that most cells away from the branching point are assigned with high confidence to one of the branches. However, cells that are equidistant from both branches have high assignment uncertainty (0.5). This is also the case for cells close to the branching location where the two branches are in close proximity. In the bottom panel of Fig. 1b, the posterior on the branching location shows there is significant uncertainty on the precise branching location. This is reflected in Fig. 1a in the branching time uncertainty (magenta). The cell assignment uncertainty is incorporated into the branching time posterior. If the branches separate quickly, the posterior branching time uncertainty is likely to be small. This reflects one of the main benefits of employing a probabilistic model to identify branching dynamics as the assignment uncertainty is considered when calculating the branching time posterior. The cell assignment is inferred in the BGP model, in contrast to the model in [12] where the assignment is assumed known.

Additional biological insights can be gleaned from the BGP method by inferring a branch order network using the posterior for each branching gene. The probability of a gene *A* branching before time *t* can be calculated using *S* samples from the branching posterior,

4 $$ P\left(\operatorname{Br}(A) < t)\right) = \frac{1}{S} \sum_{s=1}^{S} I \left(s_{A} < t \right),  $$

where Br(*A*) is the branching time of gene A and *s*_*A*_ are the posterior branching time samples. The probability of a gene *A* branching before gene *B* can be calculated similarly from each branching posterior,

5 $$ P\left(\operatorname{Br}(A) < \operatorname{Br}(B)\right) = \frac{1}{S} \sum_{s=1}^{S} I \left(s_{A} < s_{B} \right).  $$

We can infer a branch order network by computing this probability for all pairs of branching genes at the desired confidence level. In the single-cell applications we discuss later, we use this approach to construct a directed network graph of gene branching times.

We can also calculate a posterior rank for each gene with an associated confidence interval. Using samples of the posterior branching time, we can estimate quantiles of the rank distribution for each gene. This is a simple way to infer which genes branch early and which late through a probabilistic ranking. Unlike the previous approach, this does not allow pairs of genes to be compared but can provide an overall summary of the branch ordering without the need for a network analysis.

### Synthetic study

We evaluate three methods, MFA [17], the BEAM approach [3] and the BGP model, on synthetically generated data. For the synthetic study, we use Gaussian noise and therefore, we use the BEAM algorithm with a Gaussian likelihood function. MFA also assumes a Gaussian likelihood function. Data are generated from branching GPs with signal variance *σ*^2^=2, length scale *λ*=1.2 and a range of noise levels (Table 2). Samples where the functions cross after the branching point were rejected since these may be difficult for other methods, e.g. BEAM identifies the last crossing location for its fitted splines and may, therefore, identify the wrong point in a time series that crosses after branching. We address this issue in the real-data study considered in the next section but do not consider it in the synthetic benchmark. We generate *N*=150 data points (cells) with *D*=40 genes and pseudotime in a unit interval [0,1]. The genes are separated into three groups depending on their branching behaviour and time (Table 1).

**Table 1:** Synthetic gene groups

Group	Branching time	*G*
Early	0.2	10
Late	0.8	20
No branching	NA	10

Branching times and number of genes *G* for each group. All scenarios use *N*=150 cells and a total of *D*=40 genes

*NA* not applicable

**Table 2:** Synthetic study: pseudotime rank correlation to the true time for both MFA and Monocle under both scenarios

Noise	MFA	Monocle
0.001	−0.96	1.0
0.01	0.98	1.0
0.03	−0.93	1.0
0.08	−0.97	1.0
0.1	−0.97	1.0
0.2	0.91	1.0

*MFA* mixture of factor analysers

All methods were run with default parameter settings, so it may be possible to improve on their performance by tuning these parameters. For example, as in [17], we found the performance of the algorithm depended on the initialisation used. We contrast the performance of the BGP model both without and with an informative prior (80% prior probability) on cell assignment derived from the global Monocle assignment.

We first compare the pseudotime estimation accuracy of Monocle and MFA. Both methods achieve good performance as measured by the rank correlation of the estimated pseudotime to the ground truth (Table 2).

The Bayes factor of the branching GP can be used to rank the evidence of branching for each gene. Similar measures exist for MFA and the BEAM method. We first compare the three methods on their ability to discriminate branching from non-branching genes (Table 3). The metric we use is the area under the curve, which provides a reasonable measure when the numbers of positives and negatives in the ground truth are balanced. Both BEAM and BGP have higher accuracy than MFA, whose performance varies significantly. The inclusion of an informative prior improves the performance of the BGP model, resulting in consistently high performance for all noise levels. The performance of BEAM decreases with increased noise level, which is also the case for the BGP model to a lesser extent.

**Table 3:** Synthetic study: Area under the curve for detecting branching genes

Noise	MFA	BEAM	BGP	
			No prior	prior
0.001	0.30	1.00	1.00	1.00
0.01	0.65	1.00	1.00	1.00
0.03	0.77	0.96	0.98	0.99
0.08	0.82	0.93	0.86	0.92
0.1	0.77	0.82	0.81	0.96
0.2	0.77	0.74	0.86	0.84

*BEAM* branch expression analysis modelling, *BGP* branching Gaussian process, *MFA* mixture of factor analysers

**Fig. 2:**
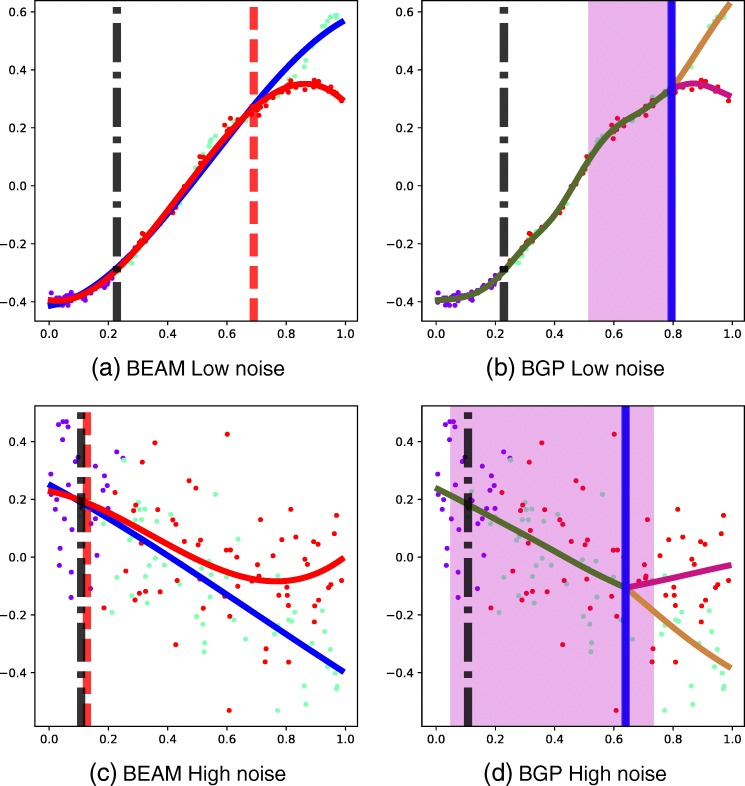
Synthetic data: example BEAM and BGP model predictions for late-branching genes. These branch at *b*=0.8. Pseudotime is shown on the horizontal axis and the gene expression is depicted on the vertical axis. The global branching time (vertical black bar) and the posterior branching time uncertainty (magenta background) are shown. The vertical red bar is the BEAM branching time estimate and the vertical blue bar the BGP estimate. Cells have been coloured by the global Monocle assignment. **a** BEAM low noise. **b** BGP low noise. **c** BEAM high noise. **d** BGP high noise. BEAM branch expression analysis modelling, BGP branching Gaussian process

We also examine the error in identifying the branching time. As MFA does not provide such an estimate, we consider only the BEAM and BGP methods. The error in estimating branching time for the BEAM and BGP methods is given in Table 4. The error for the BGP method is consistently lower than that for the BEAM method. The informative prior allows for more consistent performance of the BGP method. This method has substantial increases in accuracy in some scenarios, especially for the highest noise level (0.2), where the error is reduced from 0.15 to 0.08. The lack of robustness of the BEAM approach to high noise is demonstrated in Fig. 2. In the low-noise scenario (Fig. 2a, b), both BEAM and BGP are able to recover the gene expression branching dynamics. In the high-noise scenario (Fig. 2c, d), the global branching time is early due to the early-branching genes in the data. The later-branching gene depicted has a branching time of *b*=0.8 and the global assignment correctly separates the two branches. However, due to the high noise in the data, the spline is unable to identify the correct branching time and significantly underestimates it (Fig. 2c). In contrast, the BGP model correctly identifies the late-branching nature of the gene, despite the early global branching time and the high-noise level of the data (Fig. 2d).

**Table 4:** Synthetic study: root-mean-squared error for branching time estimation

Noise	BEAM	BGP	
		No prior	prior
0.001	0.12	0.03	0.03
0.01	0.11	0.04	0.05
0.03	0.13	0.06	0.07
0.08	0.20	0.14	0.09
0.1	0.23	0.12	0.09
0.2	0.23	0.15	0.08

Only performed on branching genes

*BEAM* branch expression analysis modelling, *BGP* branching Gaussian process

**Fig. 3:**
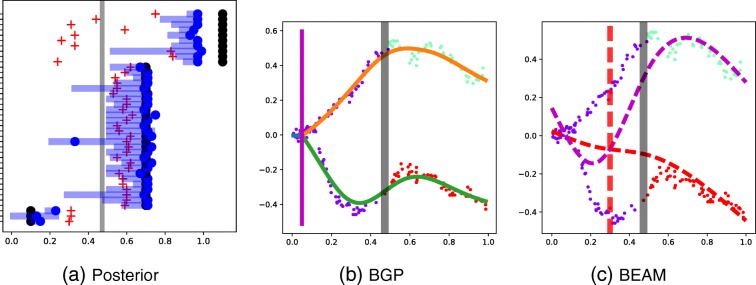
Synthetic data: fitting BGP and BEAM. The horizontal axis depicts the pseudotime. **a** The true branching times (black dots), BEAM times (red crosses), BGP mean (blue dots) and 98% credible regions for all 40 synthetic genes. **b**,**c** BGP and BEAM estimates for the same early-branching gene. Gene expression is depicted on the vertical axis. The vertical grey bar is the global branching time. The vertical red dashed bar is the BEAM branching time estimate and the vertical magenta bar the BGP estimate. Cells have been coloured by the global assignment. BEAM branch expression analysis modelling, BGP branching Gaussian process

More generally, the spline approach taken in BEAM suffers from a consistent bias in branching time estimation, which pulls all estimates towards the global branching time. To demonstrate this effect clearly, we examine an additional synthetic example with three genes branching very early (0.1), 27 genes branching late (0.7) and 10 genes not branching. We select a low noise level (0.001). Since there are many late-branching genes and few early-branching genes, the global branching time is late (Fig. 3), which clearly demonstrates the bias effect. As can be seen in Fig. 3a, the estimates for the BEAM method are biased towards the global branching time. The underestimation of branching times in BEAM for genes that branch later than the global branching time is most likely due to the spline regularisation employed by BEAM, which tends to over-smooth the spline fit. The overestimation of branching times for early-branching genes is due to the arbitrary assignment of cells prior to the global branching time, as no labels are provided by the global algorithm and no estimation is performed by the spline-fitting algorithm. See Fig. 3c for an illustrative example. The former could possibly be rectified by tuning the regularisation approach employed, but the latter is a fundamental restriction of the BEAM approach, which does not directly estimate branching assignments but uses only the globally derived label estimates. The BGP approach (Fig. 3b) does not suffer from this deficiency, as the branch assignment is performed gene by gene at the cost of increased computation time. However, the task is easily parallelisable, as each gene is treated independently.

**Fig. 4:**
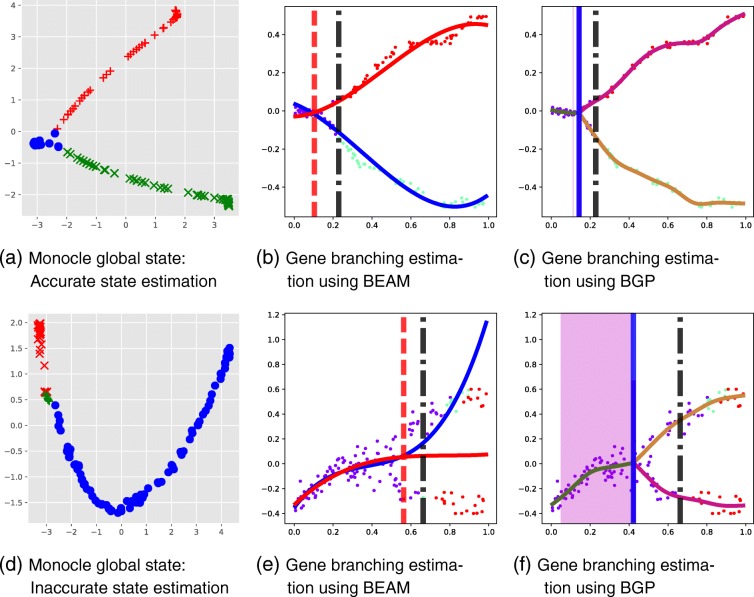
Synthetic data: effect of Monocle global state estimation on BEAM and BGP model predictions for early-branching genes. *b*=0.2. Two different examples are shown, corresponding to accurate and inaccurate state estimation by Monocle. **b**, **c**, **e**, **f** Gene expression (vertical axis) vs pseudotime (horizontal axis). The vertical black bar is the global branching time. The vertical red bar is the BEAM branching time estimate and the vertical blue bar the BGP estimate. Cells have been coloured by the global Monocle assignment. **a**, **d** The Monocle-DDRTree latent space. BEAM branch expression analysis modelling, BGP branching Gaussian process

Lastly, we examine the effect of poor state estimation on the BEAM and BGP methods (Fig. 4). The generated data sets are identical except for the level of Gaussian observation noise, whose variance is increased from *σ*^2^=0.001 (Fig. 4a) to *σ*^2^=0.03 (Fig. 4d). In Figs. 4a–c, the Monocle state estimation accurately identifies the underlying branching dynamics and both BGP and BEAM correctly estimate the branching dynamics. In Figs. 4d–f, we show an example of the effect of poor state estimation.

The state estimation correctly identifies a single branching point but the majority of cells are assigned to one of the branches (red). As one of the global branches (red) spans both gene expression branches, it is unsurprising that the spline approach fails to identify the branch location correctly and in fact overestimates the true branching time of *t*_*b*_=0.2 (Fig. 2a). The corresponding BGP inference (Fig. 4f) overcomes the errors in global state estimation and the confidence interval includes the true branching time.

The robustness of the BGP model can be understood in terms of the probabilistic nature of the model. Prior global state information is considered as well as a likelihood term that fits a branching process. Therefore, the BGP prior model incorporates the best of both worlds: inclusion of global assignment information and assessment of cell assignment based on individual gene expression. In the supplementary material (Additional file 1: Section 3), we also show the robustness of the model to non-Gaussian data, using synthetic data generated using a single-cell RNA-seq data simulator [21] to generate zero-inflated count data across a range of library sizes and dropout rates. We also find the model robust to data downsampling (Additional file 1: Section 4), which we use to speed up inference in the studies of experimental data that follow.

### Haematopoiesis and single-cell RNA-seq

We apply the BGP model on single-cell RNA-seq of haematopoietic stem cells (HSCs), which differentiate into myeloid and erythroid precursors [18]. Following the same quality control as [2], the data consists of 2312 genes and 4423 cells. We use *M*=30 inducing points in our sparse approximation and to speed up the computations further, we randomly subsample the data down to 467 cells. In [2], an analysis of this data set was performed and we use their estimation of pseudotime and cell trajectory assignment labels using the Wishbone algorithm. We found similar pseudotime and global branching assignments with Monocle 2 (vs 2.1), and those results are given in the supplementary material (Additional file 1: Section 7).

To reduce the computation required, we apply a *t*-statistic on the end states of the two branches to filter the genes that are most likely branching. We apply the BGP algorithm on the resulting set of 1072 genes. The BGP inference was performed in parallel for each gene and required approximately 2 minutes of CPU time per gene^1^.

A probabilistic model is an appropriate choice for early haematopoiesis, which has been described as a cellular continuum of low-primed HSCs [22]. The continuum contains transitory states rather than discrete progenitor cell types. Some cell state transitions and lineage combinations are more likely to occur than others. A probabilistic model such as BGP better reflects the probabilistic nature of lineage selection highlighted in [22]. In the BGP model in particular, each cell is associated with an allocation probability for each branch. The branching point can be interpreted as the earliest pseudotime from which probabilistic biases in lineage selection can be detected.

**Fig. 5:**
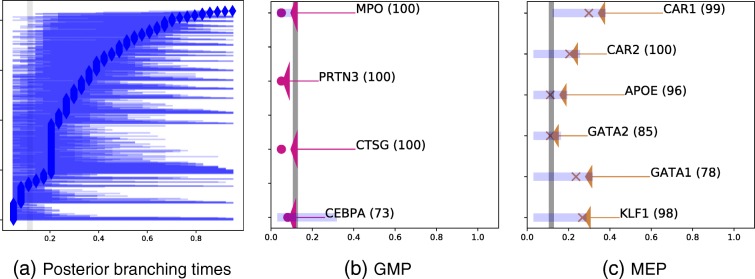
Haematopoiesis data. The horizontal axis is pseudotime in all plots. **a** Posterior branching times for 839 genes identified as branching (log Bayes factor >0) ordered by branching location. The global branching time is shown as a vertical grey bar (*b*=0.11). The vertical axis is the gene index. **b**, **c** Branching times for marker genes that have been found to show significant evidence of branching for megakaryocyte erythroid progenitors (MEPs) and granulocyte macrophage progenitors (GMPs). The colour scheme reflects which branch is upregulated after the most likely branching time. The arrows associate each gene with its most likely branching time (dot) and posterior branching confidence interval (blue region). The percentile rank in terms of the log Bayes factor is shown in parentheses; e.g. 100 means ranked in the first percentile of all 1072 genes examined. GMP granulocyte macrophage progenitor, MEP megakaryocyte erythroid progenitor

We find 839 genes out of a possible 1072 that show evidence of branching based on the Bayes factor. The posterior branching times for all branching genes are shown in Fig. 5a. Only 76 genes have a log likelihood over 50, with the majority of the genes close to the 0 threshold. In Fig. 5, we also show the branching times for 10 marker genes that have been found to show significant evidence of branching. Marker genes for megakaryocyte erythroid progenitors (MEPs) and granulocyte macrophage progenitors (GMPs) are shown. For all GMP markers, the same branch is upregulated (magenta), whereas for the MEP markers, the alternative branch is upregulated (brown). The confidence interval of branching times is also shown for each marker gene, which can be used in drawing inferences. For example, the CTSG GMP marker is predicted to branch earlier than the global branching time with high confidence, whereas the branching time of the CEBPA marker is highly uncertain.

**Fig. 6:**
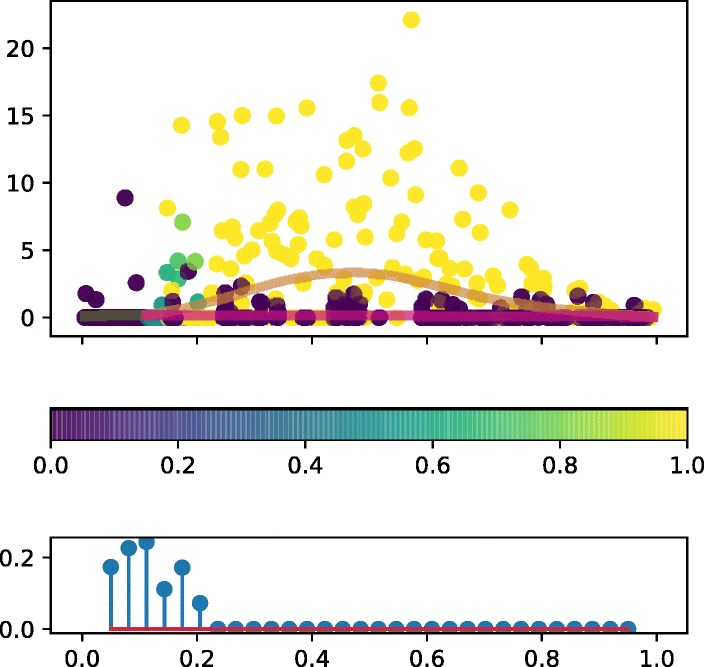
Haematopoiesis data: example of transitory gene expression for the APOE gene. Pseudotime is shown on the horizontal axis. The cell assignment uncertainty (top, vertical axis) and posterior branching time posterior (bottom, vertical axis) is shown for the BGP method. BGP branching Gaussian process

When examining the model fit for the APOE marker gene (Fig. 6), we observe a transitory gene expression for one of the branches. In particular, the expression initially increases after the branch point, peaks and decreases to the level of the other branch. The spline approach used in BEAM erroneously identifies the last intersection point as the branching point, as we show in the supplementary material (Additional file 1: Section 7), whereas the BGP approach computes a posterior over the branch locations, which quantifies the likelihood of both intersection points as branching locations.

**Fig. 7:**
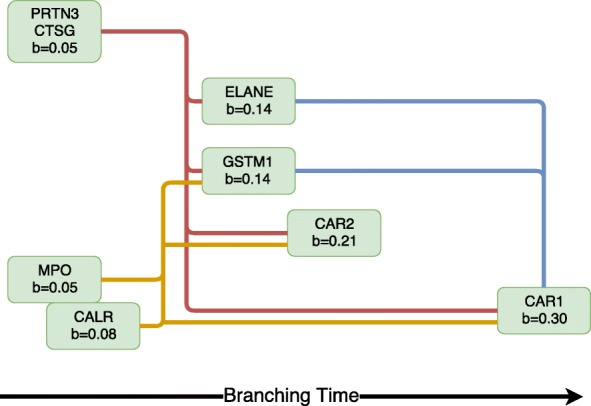
Haematopoiesis gene expression. Network of most significantly branching genes (log Bayes factor >200). The most likely branching time is given for each gene. The directed edges denote the gene branching order with a 95% confidence cut-off. The edge colours are used to group genes that have identical later branching genes. The horizontal placement of each gene is based on its most likely branching time

**Fig. 8:**
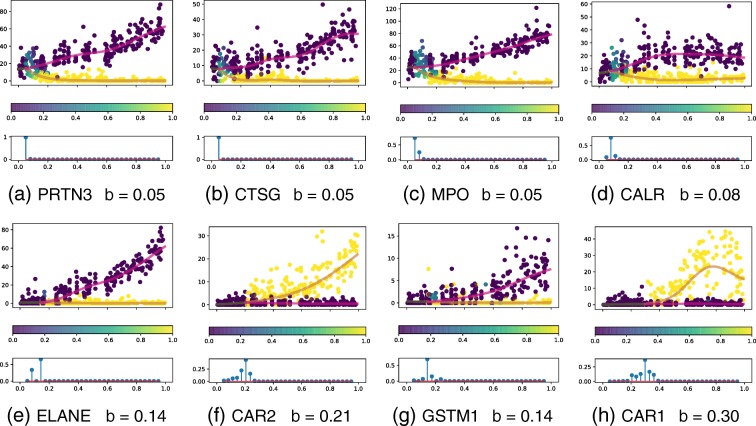
Haematopoiesis gene expression. Gene expression for most significantly branching genes (log Bayes factor >200). The bottom panel for each gene denotes the posterior branching time over the discrete set considered. **a** PRTN3 *b*=0.05. **b** CTSG *b*=0.05. **c** MPO *b*=0.05. **d** CALR *b*=0.08. **e** ELANE *b*=0.14. **f** CAR2 *b*=0.21. **g** GSTM1 *b*=0.14. **h** CAR1 *b*=0.30

We show the branching time network and gene expression profiles for the highest evidence branching genes in Figs. 7 and 8. These eight genes show very strong evidence of branching (*r*_*g*_>200).

In the network (Fig. 7), each gene is annotated with its most likely branching time and the pairwise branching time order relationships are denoted by directed edges. The posterior confidence cut-off used for the latter is 95%. For instance, both PRTN3 and MPO are found to branch before CAR1 but only the former is branching before ELANE; this can be understood by the higher uncertainty in the branching posterior of MPO (Fig. 8c). In the network we have groups genes in three distinct modules. The PRTN3 and CTSG genes (red module) branched before all other genes except CALR, i.e. ELANE, CAR1, CAR2 and GSTM1. The other group of early-branching genes, MPO and CALR (yellow module) branch before CAR1, CAR2, GSTM1 but not ELANE. Finally, the later branching group consisting of ELANE and GSTM1, branch before CAR1.

When considering more genes, looking at the entire network may be cumbersome and if the interest is solely on identifying the earliest branching genes, we can estimate the posterior rank for each gene as described in the BGP overview section. When using a lower threshold on the branching evidence (log Bayes factor >50), we find 76 genes are the earliest and latest branching genes, which are listed in the supplementary material (Additional file 1: Section 5).

This analysis demonstrates the richness of the BGP model and the range of downstream analyses afforded by the probabilistic nature of the model.

### Droplet-based single-cell RNA-seq

We further demonstrate the effectiveness of the BGP model by applying it to single-cell RNA-seq data generated using droplet barcoding [19]. Klein et al. [19] monitored the transcriptomic profiles and heterogeneity in the differentiation of mouse embryonic stem cells after leukaemia inhibitory factor withdrawal. A total of 2717 cells were profiled. Altogether, 24 175 transcripts were observed with cells captured at *t*=0, 2, 4 and 7 days. As for the haematopoiesis data, BGP inference was performed in parallel for each gene, which required approximately 2 minutes of CPU time per gene. The effect of cell cycle was removed using the scLVM approach [7] as in [1], who used this data set for their analysis of DPT.

We use the pseudotime estimated using the DPT method of [1]. The prior on the branching labels was also derived from the DPT method with a prior confidence of 99%. We examine the first dominant branching event reported by [1]. Here 915 cells were assigned to the trunk, and 1662 and 114 cells to each branch. To allow for fast computation for all genes, we subsampled the gene expression data down from 2717 to 335 cells with 81 assigned to the trunk, and 159 and 95 to each branch. This ensures the branches have roughly the same number of points. For faster computation, we analyse the top 998 genes according to the method of [23], which is available in the ScanPy software library [24].

**Fig. 9:**
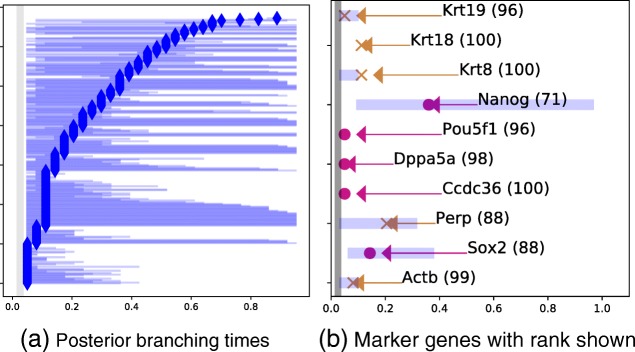
Summary plots for mouse embryonic stem cell droplet data. The horizontal axis denotes pseudotime and the vertical axis is the gene index. **a** The most likely branching time (diamonds) and confidence interval (blue region) for all genes found to show evidence of branching. **b** The posterior for selected marked genes is coloured by the branch that is enriched (brown or magenta)

A summary of the BGP findings is shown in Fig. 9. A total of 337 genes show evidence of branching and 661 do not. There is a continuum of early to late posterior branching times (Fig. 9a).

**Fig. 10:**
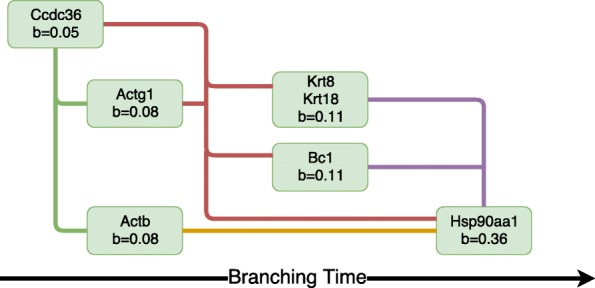
Mouse embryonic stem cell droplet data. Expression profiles for genes appearing in the branching order network (Fig. 11) and an example of a gene with higher branching uncertainty (Perp). The bottom panel in each gene expression denotes the posterior branching time over the discrete set considered. **a** Ccdc36 *b*=0.05. **b** Actg1 *b*=0.08. **c** Actb *b*=0.08. **d** Krt8 *b*=0.11. **e** Krt18 *b*=0.11. **f** Bc1 *b*=0.11. **g** Hsp90aa1 *b*=0.36. **h** Perp *b*=0.20

We also show the branching times for a selection of the genes found to be differentially expressed in [1] (Fig. 9). The percentile rank in terms of the log Bayes factor is also shown for each gene. All three epiblast markers considered (Krt8, Krt18 and Krt19) show strong evidence of branching early in pseudotime and are upregulated in the main branch (brown). They are also very highly ranked. Krt8 and Krt18 are both in the top percentile and Krt19 is in the 96th percentile. The gene expression profiles (Fig. 10) show clear early-branching times for the epiblast markers as well as for genes where the alternative branch is upregulated (e.g. Ccdc36). The branching time uncertainty is low for most marker genes, including the epiblast markers. We also show an example of transitory gene expression (Perp), where the BGP method selects the earliest intersection point as the most likely branching time and this point has a higher posterior branching time uncertainty.

**Fig. 11:**
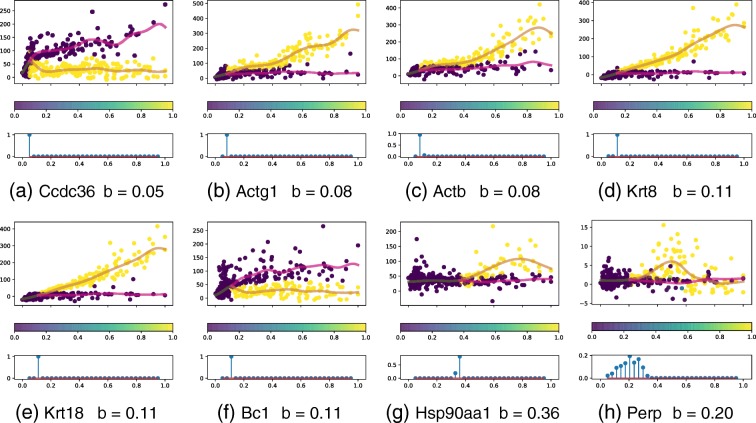
Mouse embryonic stem cell droplet data. Gene network of branching times of most significantly branching genes (log Bayes factor >500). A directed edge *A*→*B* denotes that gene *A* branches before gene *B* with a 95% probability. The edge colours are used to group genes that have identical later branching genes. The horizontal placement of each gene is based on its most likely branching time

We use the branching time posterior for each gene to estimate a branch order network of genes. For ease of presentation, we examine only the seven branching genes with the strongest evidence of branching (log Bayes factor >500). All posterior rankings with confidence greater than 95% are included in the network. We show the gene branch order network in Fig. 11 and the corresponding gene expression profiles in Fig. 10. The earliest branching gene, Ccdc36, was found to branch before all other genes in the network within the 95% confidence threshold. The Actg1 and Ccdc36 genes branch before the later branching genes Krt8, Krt18, Bc1 and Hsp90aa1. The epiblast markers Krt8 and Krt18 and the Bc1 gene branch before Hsp90aa1, which has the latest branching time (0.36). The earliest branching genes can also be found by directly computing the posterior rank for each (see section ‘Overview of BGP’). These genes are listed in the supplementary material (Additional file 1: Section 6).

## Conclusion

We have presented a flexible non-parametric probabilistic approach for robustly identifying individual gene branching times. For scalability, our model uses sparse variational inference implemented using the GPflow package [16]. The probabilistic nature of our model allows for well-defined parameter estimation via maximisation of a bound on the marginal likelihood.

The spline model used by BEAM uses global branch assignments for each cell and is, therefore, unable to accurately identify branching times earlier than the global branching time. We found that branching time estimates from this spline-based approach were generally biased towards the global branching time. In contrast, the BGP method can robustly identify branching times, as it estimates the cell branch association for each gene independently while accounting for cell assignment uncertainty in the posterior branching times. We also found the BGP approach to be robust to global state estimation errors and high noise. The BGP branching time uncertainty can also be used in a downstream analysis of the individual gene branching times, for example, ranking genes in terms of their most likely or minimum branching times.

In the BGP model, a separate assignment of cells to branches is performed for each gene, since they are treated independently. This can lead to potentially misleading results, as cells may be assigned to different branches for different genes. Therefore, to achieve good results, we use an informative prior based on the cell assignment of a global method such as Monocle [3], DPT [1] or Wishbone [2]. This gives a high prior confidence of cell assignments after the global branching point. By using this strongly informative prior, we avoid the issue of inconsistent assignments for cells with pseudotime after the global branching point. However, cell assignments can differ for genes branching prior to the global branching time and therefore, care must be taken when interpreting the results for such genes. This is an improvement over methods such as BEAM that randomly assign cells prior to the global branching time. In future work, we plan to extend the BGP model to share the cell assignment across all genes, therefore avoiding such inconsistencies and simplifying any downstream analysis.

We have also included in our comparison a probabilistic linear method [17]. The linearity allows for an efficient joint estimation of both the pseudotime and global branching structure. Although this method does not estimate gene bifurcation times, a probabilistic estimate of whether an individual gene exhibits branching behaviour is available. However, in our synthetic study, we found that the pseudotime estimation was not robust and this reduces the effectiveness of the method.

The application of the BGP method to the haematopoiesis data revealed the importance of modelling transitory gene expression, which has the potential to confuse non-probabilistic methods. The model was able to select automatically the most likely branching location, even in the presence of multiple crossing points in gene expression without the need for any post-processing heuristics such as those included in the BEAM package.

We also demonstrated the flexibility of our approach by applying it to droplet-based single-cell data using the pseudotime and branching association derived from the DPT method [1]. As the BGP approach does not rely on a particular method to estimate pseudotime and branching association, a modular approach is possible in which the best method for a given study is used. The prior uncertainty specification on the branching association allows the BGP user to quantify the expected accuracy of the global branching method.

The probabilistic nature of the BGP model allows for additional biological insights to be gained by constructing a gene branch order network and identifying early lineage priming. The former is accomplished by computing a pairwise gene probability that assesses the likelihood of a gene branching before another. The latter can be inferred by examining the gene network and identifying the earliest branching genes. We demonstrated this approach on both single-cell data sets, identifying early-branching genes and confident orderings of gene branching events.

Concurrent with our study, [25] used changepoint kernels to develop similar branching GPs to identify bifurcations in single-cell transcriptional data sets. They use a Markov chain Monte Carlo approach to estimate cell branch association and branching times. Their approach also explicitly models recombination, in which individual branches are merged, and they can jointly estimate pseudotime. However, the computational complexity of their method would make application to genome-wide inference of branching times from unlabelled data challenging and that is the motivation for our sparse inducing point variational approach.

A number of extensions of the BGP model are possible that would increase the range of possible applications. The branching kernel could be adapted to detect changepoints in time series. Whereas we have modelled a branching event as the intersection of three latent functions, a changepoint would require only two latent functions. We would also like to extend our model to non-Gaussian likelihoods, which would more accurately describe single-cell data. This would increase inference complexity but could provide better calibrated uncertainty estimates. Another useful extension would be to jointly infer pseudotime and branching behaviour, which would also improve uncertainty estimation as the uncertainty arising from the estimation of the former would be included in the posterior branching uncertainty. Extending our model to multiple branching points is straightforward from a modelling standpoint but presents a more challenging optimisation problem, for which a tree prior on the branching structure may prove helpful [26]. This extension would allow us to address the problem of selecting the correct number of branches in the global cellular branching dynamics.

## Methods

### Branching model derivation outline

We present an overview of the probabilistic model for BGP. The full model description and derivation of the variational inference lower bound is given in the supplementary material (Additional file 1: Section 2).

First, we define the Gaussian process kernel describing a branching structure. We then derive a lower bound on the model likelihood using variational inference. In the supplementary material (Additional file 1: Section 2.2), we present a formulation of a sparse inducing point approximation that allows the application of the model to large data sets. How to perform prediction on the full and sparse models is also presented in the supplementary material (Additional file 1: Section 2.3).

#### Branching kernel

To model the branching process, we specify a branching kernel that constrains the latent branching functions to intersect at the branching point. We use a modified version of the kernel proposed in [12]. The trunk *f* and branch kernel functions *g* and *h* are constrained to cross at the branching point *t*_*p*_. We place Gaussian process priors on all three functions and constrain them to intersect:

6 $$ \begin{aligned} f &\sim \mathcal{GP}(0,K), \\ g &\sim \mathcal{GP}(0,K), \\ h &\sim \mathcal{GP}(0,K), \\ f\left(t_{p}\right) &= g\left(t_{p}\right) = h\left(t_{p}\right). \end{aligned}  $$

For simplicity, the same kernel is used for all three functions although it would be straightforward to extend our framework to specify different kernels for each latent function. This would allow for instance, one branch to be modelled as a periodic function and the others as non-periodic. The extra flexibility would come at the cost of increasing the number of parameters that need to be estimated.

The resulting covariance between any two latent functions *f* and *g* constrained to cross at *t*_*p*_ is

7 $$\begin{array}{*{20}l} \Sigma &= \left(\begin{array}{ll} K_{ff} & K_{fg} \\ K_{gf} & K_{gg} \end{array} \right) \\ & = \left(\begin{array}{cc} K(\mathbf{T},\mathbf{T}) & \frac{K\left(\mathbf{T},t_{p}\right)K\left(t_{p},\mathbf{T}\right)}{K\left(t_{p},t_{p}\right)}\\ \frac{K\left(\mathbf{T},t_{p}\right)K\left(t_{p},\mathbf{T}\right)}{K\left(t_{p},t_{p}\right)} & K(\mathbf{T},\mathbf{T}) \end{array}\right). \end{array} $$

where *K*(**T**,**T**),*K*(**T**,*t*_*p*_) and *K*(*t*_*p*_,*t*_*p*_) are the kernel functions evaluated between all training data pseudotimes **T**, between the training data and branching point, and solely at the branching, point respectively.

In [12], only two latent functions were specified, a control and perturbation condition where the former spanned the branching point. In our modified formulation, three functions are used, allowing for a discontinuity in the gradient between the trunk and both branch latent functions. As an extension of our model, the derivatives of the latent functions could also be constrained to intersect at the branch point to allow for differentiable paths.

#### Full GP inference

Let $Y\in \mathbb {R}^{N}$ be the data of interest and let *M*_*f*_ be the number of functions that are dependent. We specify a set of latent functions *F* for each data point using an expanded representation of size *M*×1 where *M*=*NM*_*f*_^2^. Let *Z*∈{0,1}^*N*×*M*^ be the binary indicator matrix on the expanded representation that describes the association of each data point to a latent function. Each row of *Z* has only one non-zero entry. The model likelihood is

8 $$ p\left(Y|F,Z\right)=\mathcal{N}\left(Y|ZF,\sigma^{2}I\right).  $$

The extension to multiple independent outputs is straightforward as the likelihood factorises:

9 $$ p\left(Y|F,Z\right)=\prod_{d=1}^{D}\mathcal{N}\left(Y_{d}|ZF_{d},\sigma^{2}I\right),  $$

where *Y*_*d*_ denotes the *N*×1 column vector of observations for output *d* and similarly *F*_*d*_ denotes the *M*×1 column vector of latent function values. We omit the multiple output case from the derivation below for clarity.

As in [13], we place a categorical prior on the indicator matrix *Z* and a GP prior on the latent functions *F*. Note that the latter does not factorise as in [13], as we assume the latent functions are dependent:

10 $$\begin{array}{*{20}l} p(Z) & =\prod_{n=1}^{N}\prod_{m=1}^{M}\left[\Pi\right]_{n,m}^{[Z]_{nm}}, \end{array} $$

11 $$\begin{array}{*{20}l} p(F) & =\mathcal{N}\left(0,K\right), \end{array} $$

where for the multinomial distribution we have $\sum _{m=1}^{M}\left [\Pi \right ]_{nm}=1$ and *K* is the GP kernel^3^.

The log likelihood is not analytically tractable, as it involves integrating out the indicator matrix *Z*:

12 $$ \log p\left(Y|F\right)=\log\int p\left(Y,Z|F\right) \mathrm{d} Z.  $$

We proceed to compute a lower bound using Jensen’s inequality:

13 $${} \begin{aligned} \log p\left(Y|F\right) &= \log\int p\left(Y,Z|F\right)\frac{q(Z)}{q(Z)} \,\mathrm{d} Z \\ & =\mathbb{E}_{q(Z)}\left[\log p\left(Y,Z|F\right)\right]-\mathbb{E}_{q(Z)}\left[\log q(Z)\right]. \end{aligned}  $$

From the mean-field assumption, the latent functions *F* are independent of the association indicators *Z*:

14 $$ q\left(Z,F\right)=q(Z)q(F).  $$

The log likelihood term is

15 $$ \begin{aligned} \log\mathcal{N}\left(Y|ZF,\sigma^{2}I\right) = &- \frac{N}{2}\log(2\pi)-\frac{N}{2}\log\left(\sigma^{2}\right)\\ &- \frac{1}{2\sigma^{2}}\left(Y-ZF\right)^{T}\left(Y-ZF\right). \end{aligned}  $$

Taking the expectation with respect to the variational distribution *q*(*Z*):

16 $${} {\begin{aligned} \mathbb{E}_{q(Z)}\left[\log\mathcal{N}\left(Y|ZF,\sigma^{2}I\right)\right] = &- \frac{N}{2}\log(2\pi)-\frac{N}{2}\log\left(\sigma^{2}\right)\\ &- \frac{1}{2\sigma^{2}}\left(Y^{T}Y+F^{T}A F-2F^{T}\Phi^{T}Y\right), \end{aligned}}  $$

where we have defined

$$\begin{array}{*{20}l} \Phi & \triangleq \mathbb{E}_{q(Z)}(Z), \\ A & \triangleq \mathbb{E}_{q(Z)}\left(Z^{T}Z\right), \end{array} $$

and the variational approximation is

17 $$ q(Z)=\prod_{n,m}\Phi_{n,m}^{Z_{n,m}}.  $$

This encodes the mean-field assumption as we assume the posterior indicators factorise.

The second-order expectation for *A* is

18 $$\begin{array}{*{20}l} \left[A_{i,j}\right] &= \mathbb{E}_{q(Z)}\left[\sum_{n}z_{n,i}z_{n,j}\right]  \\ &=\left[\sum_{n}\Phi_{n,i}\right]\delta_{i,j}, \end{array} $$

where *z*_*i*_ the *N*×1 indicator vector for latent function *i*=*m*.

The KL divergence term is computable as

$$\operatorname{KL}\left[q(Z) \mid\mid p(Z)\right] = \sum_{n,m}\Phi_{n,m}\log\left(\frac{\Phi_{n,m}}{\left[\Pi\right]_{n,m}}\right). $$

Our bound is, therefore,

$$\log p\left(Y|F\right)\geq L_{1}, $$

where we have defined

19 $${} {\begin{aligned} L_{1}\triangleq -\frac{N}{2}\log\left(2\pi\sigma^{2}\right) &- \operatorname{KL}\left[q(Z) \mid\mid p(Z)\right]\\ &- \frac{(1)}{2\sigma^{2}}\left(Y^{T}Y+F^{T}AF-2F^{T}\Phi^{T}Y\right). \end{aligned}}  $$

We proceed to integrate out the latent functions *F* to obtain the variational collapsed bound:

20 $$ \begin{aligned} \log p(Y) & =\log\int p\left(Y|F\right)p(F) \mathrm{d} F \\ & \geq\log\int\exp\left[L_{1}\right]p(F) \mathrm{d} F. \end{aligned}  $$

This bound holds because *L*_1_ is a bound to log*p*(*Y*|*F*) and the exponent function is monotonic. More details can be found in [27].

Setting the prior on the latent function as a GP, $\log p(F) = \log \mathcal {N}(F|0,K)$, and substituting (19) into (20) results in the collapsed bound

21 $$ \begin{aligned} L_{2}\triangleq & -\frac{N}{2}\log(2\pi\sigma^{2})-\frac{1}{2\sigma^{2}}Y^{T}Y-\frac{1}{2}\log|K| \\ & \qquad - \frac{1}{2}\log\left|A\sigma^{-2}+K^{-1}\right| \\ & \qquad + \frac{1}{2}\sigma^{-4}Y^{T}\Phi\left(A\sigma^{-2}+K^{-1}\right)^{-1}\Phi^{T}Y \\ & \qquad - \operatorname{KL}\left[q(Z) \mid\mid p(Z)\right].  \end{aligned}  $$

We can also derive this bound when using an inducing point approximation. This allows the algorithm to scale up to larger data sets. The full derivation is given in the supplementary material (Additional file 1: Section 2).

## Additional file

Additional file 1 Supplementary material for BGP: identifying gene-specific branching dynamics from single-cell data with a branching Gaussian process. This also contains additional results for both the synthetic and single-cell data sets. (PDF 1206 kb)

## Footnotes

1 Time measured on a MacBook Pro with 2.2GHz quad-core Intel Core i7 and 16GB of DDR3L onboard memory.

2 This expanded representation allows for efficient recomputation of the marginal likelihood for different branching times.

3 For simplicity, we assume the same kernel for every output and latent trajectory function. Removing this restriction does not affect the derivation but will increase the inference complexity.
