# Supplementary material for: BGP: identifying gene-specific branching dynamics from single-cell data with a branching Gaussian process
Source: Genome Biol. 2018 May 29;19:65. doi: 10.1186/s13059-018-1440-2 (PMC5975664; doi:10.1186/s13059-018-1440-2)
Supplement: Supplementary file 1 — Supplementary material for BGP: identifying gene-specific branching dynamics from single-cell data with a branching Gaussian process. This also contains additional results for both the synthetic and single-cell data sets. (PDF 1206 kb) [file 13059_2018_1440_MOESM1_ESM.pdf]

Supplementary material for BGP: identifying gene-specific branching dynamics from single cell data with a branching Gaussian process

Alexis Boukouvalas[1], James Hensman[2] and Magnus Rattray[1]

[1]Division of Informatics, Imaging and Data Sciences Faculty of Biology, Medicine and Health
University of Manchester
[2]prowler.io

April 28, 2018

Contents

1 Introduction **1**

2 Detailed branching model derivation **1**
2.1 Full GP inference 1
2.2 Sparse GP inducing point approximation 3
2.3 Prediction 4

3 Synthetic data: simulating single cell count data **4**

4 Synthetic data: Robustness to sparsity and subsampling **4**

5 Hematopoiesis gene network **6**

6 Mouse embryonic stem cell droplet gene network **6**

7 Hematopoiesis single cell RNA-seq using Monocle version 2.1 **6**

1 Introduction

In Section 2 we provide full details on the BGP model derivation. In Sections 3-4 we present additional experiments on synthetic data that demonstrate the robustness of the BGP model to different dropout rates on count data and the effect of the sparsity approximation and downsampling rates on model accuracy. In Section 7 we show a study on the hematapoiesis data using a Monocle to set the pseudotime and branching prior and compare to BEAM.

2 Detailed branching model derivation

We present in detail the probabilistic model description of the branching Gaussian process. We derive a lower bound on the model likelihood using variational inference techniques. Lastly we present a formulation of a sparse inducing point approximation that allows the application of the model to large datasets. We also discuss how to calculate the bound in a numerically stable manner and how to perform prediction on our model.

2.1 Full GP inference

Let $Y \in \mathbb{R}^N$ be the data of interest and let M_f be the number of functions that are dependent. We specify a set of latent functions F for each data point of size $M \times 1$ where $M = NM_f$[1]. Let $Z \in \{0,1\}^{N \times M}$ the binary indicator matrix which describes the association of each data point to a latent function. Each row of Z has only one non-zero entry. The model likelihood is

$$p(Y|F,Z) = \mathcal{N}\left(Y|ZF, \sigma^2 I\right) \ . \quad (1)$$

The extension to multiple independent outputs is straightforward as the likelihood factorizes

$$p(Y|F,Z) = \prod_{d=1}^{D} \mathcal{N}\left(Y_d|ZF_d, \sigma^2 I\right) \ , \quad (2)$$

where Y_d denotes the $N \times 1$ column vector of observations for output d and similarly F_d denotes the $M \times 1$ column vector of latent function values. We omit the

[1]This expanded representation allows for efficient recomputation of the marginal likelihood for different branching times.

multiple output case from the derivation below for clarity.

As in ? we place a categorical prior on the indicator matrix Z and a GP prior on the latent functions F. Note that the latter does not factorize as in ? as we assume the latent functions are dependent:

$$p(Z) = \prod_{n=1}^{N} \prod_{m=1}^{M} [\Pi]_{n,m}^{[Z]_{nm}} \ , \tag{3}$$

$$p(F) = \mathcal{N}(0, K) \ , \tag{4}$$

where for the multinomial distribution we have $\sum_{m=1}^{M} [\Pi]_{nm} = 1$ and K is the GP kernel[2].

The log likelihood is not analytically tractable as it involves integrating out the indicator matrix Z

$$\log p(Y|F) = \log \int p(Y, Z|F)\, dZ \ . \tag{5}$$

We proceed to compute a lower bound using Jensen's inequality

$$\begin{aligned}\log p(Y|F) &= \log \int p(Y, Z|F) \frac{q(Z)}{q(Z)} dZ \\ &= \log \left(\mathbb{E}_{q(Z)} \left[\frac{p(Y, Z|F)}{q(Z)} \right] \right) \\ &\geq \mathbb{E}_{q(Z)} \left[\log \frac{p(Y, Z|F)}{q(Z)} \right] \\ &= \mathbb{E}_{q(Z)} [\log p(Y, Z|F)] - \mathbb{E}_{q(Z)} [\log q(Z)] \ .\end{aligned} \tag{6}$$

The last equation is usually presented in terms of a likelihood term and the tractable KL term

$$\begin{aligned}\log p(Y|F) \geq\ & \mathbb{E}_{q(Z)} [\log p(Y|F, Z) - \log p(Z)] \\ & - \mathbb{E}_{q(Z)} [\log q(Z)] \\ & = \mathbb{E}_{q(Z)} [\log p(Y|F, Z)] \\ & - \mathbb{E}_{q(Z)} [\log q(Z) - \log p(Z)] \\ & = \mathbb{E}_{q(Z)} [\log p(Y|F, Z)] \\ & - KL [q(Z) || p(Z)] \ ,\end{aligned} \tag{7}$$

where

$$q(Z, F) = q(Z)\, q(F) \ , \tag{8}$$

as by a mean-field assumption the latent functions F are independent of the association indicators Z. The log likelihood term is

$$\begin{aligned}\log \mathcal{N}\left(Y|ZF, \sigma^2 I\right) =& -\frac{N}{2}\log(2\pi) - \frac{N}{2}\log\left(\sigma^2\right) \\ & - \frac{1}{2\sigma^2}(Y - ZF)^T (Y - ZF) \ .\end{aligned} \tag{9}$$

Taking the expectation with respect to the variational distribution $q(Z)$

$$\begin{aligned}& \mathbb{E}_{q(Z)} \left[\log \mathcal{N}\left(Y|ZF, \sigma^2 I\right)\right] \\ & = -\frac{N}{2}\log(2\pi) - \frac{N}{2}\log\left(\sigma^2\right) \\ & - \frac{1}{2\sigma^2}\left(Y^T Y + F^T A F - 2F^T \Phi^T Y\right) \ ,\end{aligned} \tag{10}$$

where we have defined

$$\Phi \triangleq \mathbb{E}_{q(Z)}(Z) \ ,$$

$$A \triangleq \mathbb{E}_{q(Z)}\left(Z^T Z\right) \ ,$$

and the variational approximation is

$$q(Z) = \prod_{n,m} \Phi_{n,m}^{Z_{n,m}} \ . \tag{11}$$

This encodes the mean-field assumption where we assume the posterior indicators factorize.

The second order expectation for A can be derived as follows: let z_i the $N \times 1$ indicator vector for latent function $i = m$. We then have

$$\begin{aligned}[A_{i,j}] &= \mathbb{E}_{q(Z)} \left[\sum_n z_{n,i} z_{n,j}\right] \\ &= \mathbb{E}_{q(Z)} \left[\sum_n z_{n,i} z_{n,j}\right](1 - \delta_{i,j}) \\ &+ \mathbb{E}_{q(Z)} \left[\sum_n z_{n,i}^2\right]\delta_{i,j} \\ &= \mathbb{E}_{q(Z)} \left[\sum_n z_{n,i} z_{n,j}\right](1 - \delta_{i,j}) \\ &+ \mathbb{E}_{q(Z)} \left[\sum_n z_{n,i}\right]\delta_{i,j} \\ &= \left[\sum_n \Phi_{n,i}\right]\delta_{i,j} \ .\end{aligned} \tag{12}$$

The third step follows from the fact the $z_{n,i}$ is binary and hence $z_{n,i}^2 = z_{n,i}$ and we have used the notation $\delta_{i,j}$ to denote the delta function which is 1 when $i = j$ and 0 otherwise. The first term vanishes because

[2]For simplicity we assume the same kernel for every output and latent trajectory function. Removing this restriction does not affect the derivation but will increase the inference complexity.

cells cannot be assigned to more than one branch, and therefore $z_{n,i}z_{n,j} = 0$ when $i \neq j$. In matrix notation the expectation is

$$A = \mathrm{diag}\left(\left[\sum_n \Phi_{n,i}\right]_{i=1}^{M}\right) ,$$

where diag denotes the diagonalisation of a vector and $[.]_{i=1}^{M}$ the construction of an M dimensional vector.

The KL divergence term is computable as

$$KL\left[q\left(Z\right)||p\left(Z\right)\right] = \sum_{n,m} \Phi_{n,m} \log\left(\frac{\Phi_{n,m}}{[\Pi]_{n,m}}\right) .$$

Our bound is therefore

$$\log p\left(Y|F\right) \geq L_1 ,$$

where we have defined

$$L_1 \triangleq -\frac{N}{2}\log(2\pi\sigma^2) - KL\left[q\left(Z\right)||p\left(Z\right)\right] - \frac{}{2\sigma^2}\left(Y^T Y + F^T A F - 2F^T \Phi^T Y\right) . \quad (13)$$

We proceed to integrate out the latent functions F to obtain the variational collapsed bound

$$\log p\left(Y\right) = \log \int p\left(Y|F\right) p\left(F\right) dF \geq \log \int \exp\left[L_1\right] p\left(F\right) dF . \quad (14)$$

This bound holds because L_1 is a bound to $\log p\left(Y|F\right)$ and the exponent function is monotonic. More details can be found in **?**.

Setting the prior on the latent function as a GP $\log p\left(F\right) = \log \mathcal{N}(F|0, K)$ and substituting (13) into (14) results in the collapsed bound

$$L_2 \triangleq -\frac{N}{2}\log(2\pi\sigma^2) - \frac{1}{2\sigma^2}Y^T Y - \frac{1}{2}\log|K| - \frac{1}{2}\log\left|A\sigma^{-2} + K^{-1}\right| + \frac{1}{2}\mu_F^T\left(A\sigma^{-2} + K^{-1}\right)^{-1}\mu_F - KL\left[q\left(Z\right)||p\left(Z\right)\right] . \quad (15)$$

where we have $q\left(F\right) = \mathcal{N}\left(\mu_F, \Sigma_F\right)$

$$\Sigma_F = \left(A\sigma^{-2} + K^{-1}\right)^{-1} , \quad \mu_F = \left(A\sigma^{-2} + K^{-1}\right)^{-1}\sigma^{-2}\Phi^T Y . \quad (16)$$

Expanding the quadratic term with the mean expression:

$$L_2 \triangleq -\frac{N}{2}\log(2\pi\sigma^2) - \frac{1}{2\sigma^2}Y^T Y - \frac{1}{2}\log|K| - \frac{1}{2}\log\left|A\sigma^{-2} + K^{-1}\right| + \frac{1}{2}\sigma^{-4}Y^T\Phi\left(A\sigma^{-2} + K^{-1}\right)^{-1}\Phi^T Y - KL\left[q\left(Z\right)||p\left(Z\right)\right] . \quad (17)$$

2.2 Sparse GP inducing point approximation

To speed up inference we use a sparse inducing point approximation. This allows the algorithm to scale linearly with the number of training points. The number of inducing points is specified by the user and determines the trade-off between model accuracy and runtime; decreasing the number of inducing points will reduce runtime but will increase the approximation error.

The inducing points are treated as additional variational parameters which are optimised. The model specification is the same as before:

$$p\left(Y|F, Z\right) = \mathcal{N}\left(Y|ZF, \sigma^2 I\right) . \quad (18)$$

We introduce the inducing points u and explicitly specify their conditional relationship to the latent noise-free data f

$$p\left(f|u\right) = N\left(f|K_{fu}K_{uu}^{-1}u, K_{ff} - K_{fu}K_{uu}^{-1}K_{uf}\right) \quad (19)$$

$$p\left(u\right) = \mathcal{N}\left(u|0, K_{uu}\right) . \quad (20)$$

We now introduce the sparse GP approximation:

$$q\left(f, u, Z\right) = q\left(Z\right) p\left(f|u\right) q\left(u\right) .$$

This is similar to the mean field approximation for the full model (Equation (8)) but we have introduced an additional mean-field factorisation $q\left(F\right) = p\left(f|u\right) q\left(u\right)$.

The derivation proceeds as before with the resulting bound

$$L_s \triangleq -\frac{N}{2}\log(2\pi\sigma^2) - \frac{1}{2\sigma^2}Y^T Y - \boldsymbol{\frac{1}{2}\log|P|} \boldsymbol{- \frac{1}{2\sigma^2}\mathrm{tr}\left(AK_{ff}\right)} \boldsymbol{+ \frac{1}{2}c^T c + \frac{1}{2\sigma^2}\mathrm{tr}\left(AK_{fu}K_{uu}^{-1}K_{uf}\right)} - KL\left[q\left(Z\right)||p\left(Z\right)\right] , \quad (21)$$

where the terms in bold are different compared to the full model bound (Equation (17)). In the bound above we have defined

$$\begin{aligned} P &\triangleq I + L^{-1} K_{uf} A K_{fu} L^{-T} \sigma^{-2} \ , \\ K_{uu} &\triangleq LL^T \ , \\ c &\triangleq R^{-1} L^{-1} K_{uf} \Phi^T Y \sigma^{-2} \ , \\ P &\triangleq RR^T \ . \end{aligned}$$

2.3 Prediction

In the full model the predictive posterior a new point f_* is

$$p(f_*|y) = p(f|y)\, p(f_*|f) \ .$$

The training data posterior $p(f|y)$ is approximated by $q(f)$. To predict at a single f_* point we integrate over the latent functions values at the training data f,

$$\begin{aligned} p(f_*|y) &= \int p(f_*|f)\, p(f|y)\, df \\ &\approx \int p(f_*|f)\, q(f)\, df \ . \end{aligned}$$

where $p(f_*|f) = N\left(f_*|K_{*f}K_{ff}^{-1}f, K_{**} - K_{*f}K_{ff}^{-1}K_{f*}\right)$ is the usual GP predictive density and $q(f) = N(f|\mu_F, \Sigma_F)$ as defined in Equation (16). The latter integral is tractable. Therefore there is no further approximation required past the original mean-field approximation (Equation (8)).

Similarly for the sparse approximation

$$\begin{aligned} p(f_*|y) &= \int p(f_*|f,u)\, p(f,u|y)\, df du \\ &\approx \int p(f_*|f,u)\, q(u)\, p(f|u)\, df du \\ &= \int p(f_*|u)\, q(u)\, du \ . \end{aligned}$$

3 Synthetic data: simulating single cell count data

We use the Splatter library (?) to simulate realistic single cell gene expression count data. We consider a range of different library sizes that have correspondingly higher dropout rates as the library size is reduced.

Different sequencing depths are simulated using a different mean value for the library size log-normal distribution; specifically we use 10 different levels (4 to 14). The scale parameter is kept fixed at the default value (0.1). Each dataset consists of 20 genes and 200 cells where half the genes are branching. The dropout rate at each sequencing depth for all branching genes is shown in Table 1. We first note that the mean dropout rate drops considerably from 58% at the lowest library depth to 0 at the highest. We also note there is a large variation of dropout; for example at mean library size 7, one branching genes has 47% dropout rate.

We fit the BGP model on the log transformed count data and examine its performance on identifying branching genes and on estimating the branching location. As we see in Figure 2 (a), perfect identification of branching genes (AUC 1.00) is achieved when the library mean is greater or equal to 7. We also observe a decrease in the root mean squared error (RMSE) of the estimated branching location to the true branching time at the same level. This suggests the BGP model performs well at this and higher library sizes.

The model still achieves relatively high AUC (>0.84) at the lower library sizes suggesting the ability of the BGP model to correctly identify branching genes is fairly robust. Pinning down the exact branching time is a more challenging task and at the lowest library level (4) the model's has high RMSE (0.40).

We also show two examples of the data and the BGP fit for library sizes 6 and 7 in Figure 1. The higher dropout rate for the former results in higher posterior branching uncertainty (more diffuse bottom stem plots) whereas for the latter the branching posterior is peaked at the mode.

4 Synthetic data: Robustness to sparsity and subsampling

Using the same synthetic data as in the paper, we examine the robustness of the BGP model to the number of inducing points used in the sparse approximation and to downsampling the data which we use to reduce the training times.

We have also examined the effect of varying the number of inducing points used in the sparse approximation. In all the results presented in the paper we have used $M = 20$ inducing points and we find that the model is fairly robust to this setting; we find a perfect classification of branching vs non-branching genes (AUC=1.00) for all values we have considered $M = [10, 20, 30, 40, 50]$. Our sparse inducing point approach relies on the variational free energy approximation which has been shown to approximate better the

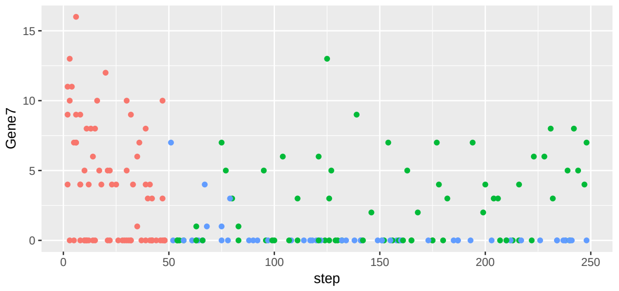

(a) Library size 6, dropout 0.59, raw data

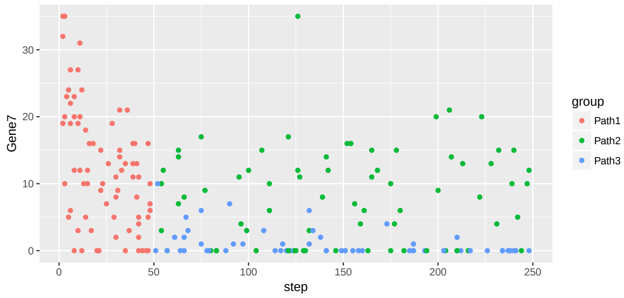

(b) Library size 7, dropout 0.33, raw data

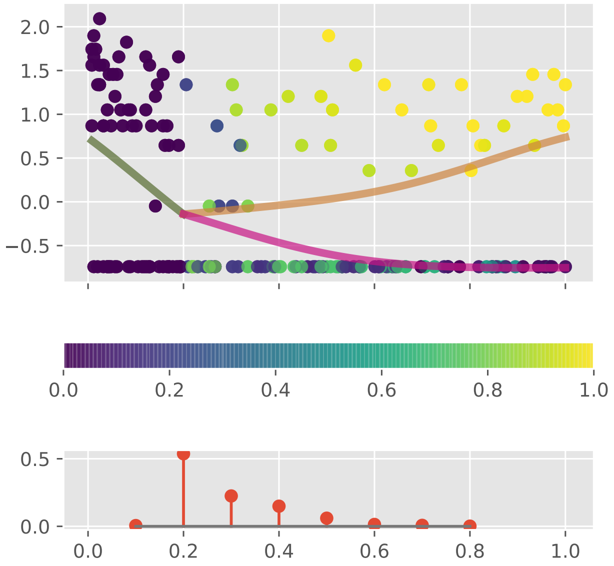

(c) Library size 6, dropout 0.59, BGP fit

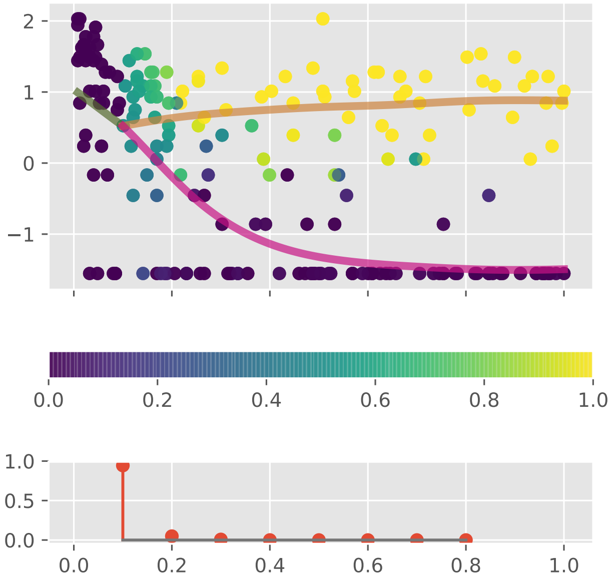

(d) Library size 7, dropout 0.33, BGP fit

Figure 1: Synthetic count data: Two branching genes sampled with different library sizes resulting in different dropout rates.

Table 1: Dropout rates for synthetic single cell count data. The dropout rate for each of the 10 branching genes is shown. Similar values are obtained for the non-branching genes but are omitted for brevity.

Library log mean	Mean dropout	Dropout rate for each gene									
4	0.58	0.39	0.61	0.30	0.26	0.42	0.88	0.91	0.72	0.48	0.84
5	0.42	0.14	0.45	0.13	0.14	0.24	0.81	0.79	0.53	0.23	0.71
6	0.26	0.14	0.22	0.08	0.10	0.08	0.66	0.59	0.24	0.11	0.42
7	0.15	0.04	0.17	0.03	0.06	0.05	0.47	0.33	0.12	0.05	0.18
8	0.09	0.01	0.10	0.01	0.01	0.03	0.29	0.23	0.07	0.01	0.12
9	0.04	0.00	0.04	0.00	0.01	0.01	0.17	0.10	0.04	0.00	0.04
10	0.02	0.00	0.01	0.00	0.01	0.01	0.09	0.07	0.01	0.00	0.01
11	0.01	0.00	0.02	0.00	0.00	0.00	0.03	0.03	0.01	0.00	0.01
12	0.00	0.00	0.00	0.00	0.00	0.00	0.03	0.01	0.01	0.00	0.00
13	0.00	0.00	0.00	0.00	0.00	0.00	0.01	0.00	0.00	0.00	0.01
14	0.00	0.00	0.00	0.00	0.00	0.00	0.01	0.00	0.00	0.00	0.00

full GP posterior as the number of inducing points is increased (?). However as the number of inducing points is increased, the computational time required grows quadratically, due to the matrix inversion required to compute the marginal likelihood lower bound which requires $O(M^2N)$ where N the total data size. We therefore use as few inducing points as possible to minimise the computational time whilst also allowing for robust approximation of the full GP posterior; in our single cell case studies, which are one-dimensional regression problems with long length-scales, where the function changes smoothly, we have found a small number of inducing points (~ 20) is usually sufficient.

We also examine the effect on model inference of reducing the data size from 150 points to 120, 100, 75 and 50 points. As in the paper, the downsampling is not random to ensure we have data points from the all three latent functions; in particular we sample 1/5 of points of trunk, and 2/5 of each global branch.

Computing the area under the curve metric to evaluate the ability of the model to identify branching genes, we also find perfect classification (auc=1.00) for all sampling levels. We find therefore the model is fairly robust when used to identify branching from non-branching genes; this conclusion agrees with the previous section which examined robustness when fitting the model to single cell count data with different library sizes.

Computing the root mean squared of the most likely branch location to the true branching point for each gene, we find consistently small error levels (< 0.04) that steadily decrease for increased sampling rates (Figure 3).

5 Hematopoiesis gene network

Using the posterior samples, we compute the median rank for each of the 839 genes found to show evidence of branching out of 1072 candidate genes. We show the ten earliest and latest branching genes in Table 2. Unlike the network analysis presented in the paper this approach does not consider the gene pairwise ordering but is simpler and can be used to identify the earliest branching genes.

6 Mouse embryonic stem cell droplet gene network

We show additional results from our analysis of the droplet barcoding data (?) used in the paper. Using the posterior samples, we compute the median rank for each gene (Table 3). We show the ten earliest and latest branching genes.

7 Hematopoiesis single cell RNA-seq using Monocle version 2.1

Version 2.1 of Monocle produce good agreement with the Wishbone approach (?) with good agreement in the estimated pseudotime (rank correlation 0.92) and significant overlap in branching assignments. However the latest version of Monocle version 2.5.2 produces a different assignment which although qualitatively similar has a smaller rank correlation (0.11). In this section we present the results when using the older Monocle version.

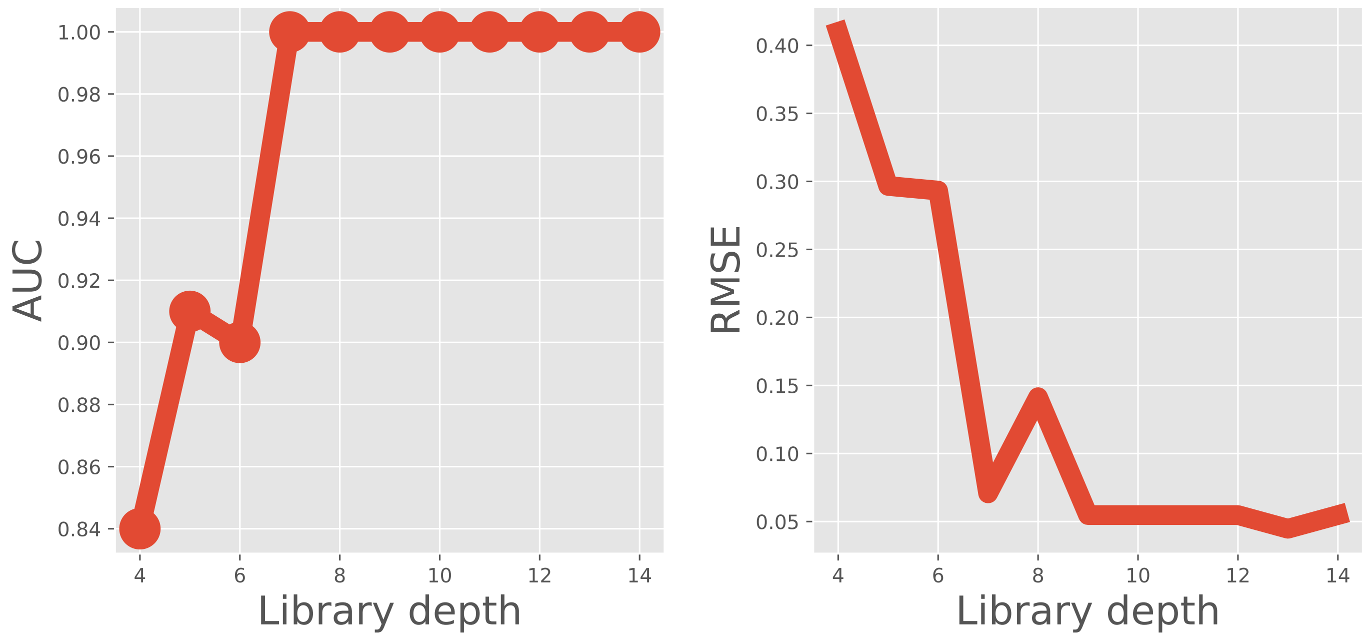

(a) Area under the curve: how well are branching genes identified?

(b) RMSE: how well is the branching point identified?

Figure 2: Synthetic count data: Performance of BGP model to identify branching genes (AUC) and branching times (RMSE) for different library sizes.

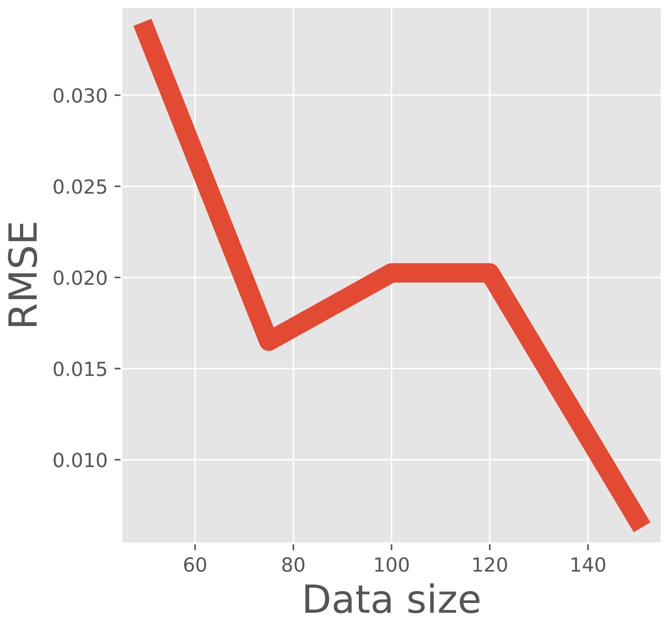

Figure 3: Synthetic Gaussian data: Performance of BGP model to identify branching times (RMSE) across different data sizes corresponding to different downsampling rates. The full data consists of 150 points.

The single-cell RNA-seq hematopoietic stem cell data was obtained from **?**. The data consists of 4423 cells. We applied the Monocle 2 algorithm (**?**) and obtained the latent space depicted in Figure 4. Numerous minor branching events were identified by Monocle which is typical when no marker genes are used. We filtered out all cells assigned to the minor branching events resulting in a set of 3854 cells that were assigned to the main branching event. We removed all genes in which more than 80% of cells had zero count resulting in a set of 1343 genes that were examined in the branching analysis. Finally, to speed up computation we randomly selected a set of 900 cells and used $M = 30$ inducing points in our sparse BGP model.

We apply the BGP model on single-cell RNA-seq of hematopoietic stem cells (HSC) differentiating into myeloid and erythroid precursors (**?**). The data processing steps are described in the Methods section. The root state was selected using marker genes for common myeloid progenitors (CMP), megakaryocyte-erythroid progenitors (MEP) and granulocyte-macrophage progenitors (GMP). The two branches are clearly distinguishable in the latent space (Figure 4). The common myeloid progenitor FLT3 is highly expressed in the root of the tree whereas the MEP marker KLF1 and GMP

Table 2: Median rank and 95% confidence interval rates for the 839 branching genes in the hematopoiesis single cell count data (log Bayes factor ¿ 0). The 20 earliest and 10 latest branching genes are shown sorted by the median rank.

	Median rank	95%	
CTSG	30	1	72
PRTN3	31	1	76
IRF8	31	1	118
F13A1	31	1	128
EMB	31	1	119
GPR56	31	1	122
PTPRCAP	31	1	127
LGALS1	31	1	119
LY86	31	1	126
TYROBP	31	1	121
P4HB	31	1	116
MPO	32	1	130
LY6C2	32	1	161
TIFAB	32	1	155
HP	33	1	191
TSPO	33	1	161
SEPX1	33	1	165
HSP90B1	33	1	231
SLPI	33	1	152
PKM2	34	1	307
...			
ADD2	819	728	830
PARVB	821	719	834
BETA-S	823	802	834
ARHGEF12	824	795	834
HEBP1	828	820	835
SEC14L2	829	809	836
SIGLECG	829	817	836
SPNB1	830	808	836
CCDC74A	834	815	839
HBA-A2	836	830	839

Table 3: Median rank and 95% confidence interval rates for the top 62 branching genes in the droplet single cell count data. The 20 earliest and 10 latest branching genes are shown ordered by the median rank.

	Median rank	95%	
Csrp1	22	1	75
Mt1	22	1	52
Krt19	22	1	53
Pou5f1	22	1	52
Malat1	22	1	52
Wbp5	22	1	66
Tor4a	22	1	52
Chchd10	22	1	108
Dppa5a	22	1	52
Hist1h2ao	22	1	52
Pbld1	22	1	52
Nkain3	22	1	53
Abcd4	22	1	63
Mif	22	1	107
Mkrn1	22	1	70
Ccdc36	22	1	52
L1td1	23	1	193
Gm6083	23	1	120
Hdgf	23	1	278
Hspa5	23	1	210
...			
Mdh2	304	143	337
Ccdc93	305	112	335
Pgk1	307	296	327
Suclg1	309	250	323
Ap2b1	310	75	321
Pkp2	313	302	323
Mark3	315	283	330
Slc25a4	317	307	333
Haus4	325	317	331
H19	330	323	335

marker MPO are expressed in each branch respectively. We also applied the Wishbone approach (?) and the two approaches have good agreement in the estimated pseudotime with a high rank correlation (0.92) and significant overlap in branching assignments.

A probabilistic model is an appropriate choice for early hematopoiesis which has been described as a cellular continuum of low-primed HSCs (?). The continuum contains transitory states rather than discrete progenitor cell types with some cell state transitions and lineage combinations more likely to occur than others. A probabilistic model such as BGP better reflects the probabilistic nature of lineage selection highlighted in ?. In the BGP model in particular, each cell is associated with an allocation probability for each branch. The branching point can be interpreted as the earliest pseudotime from which probabilistic biases in lineage selection can be detected.

We find 737 genes out of a possible 1343 that show evidence of branching based on the log Bayes factor. The posterior branching times for all branching are

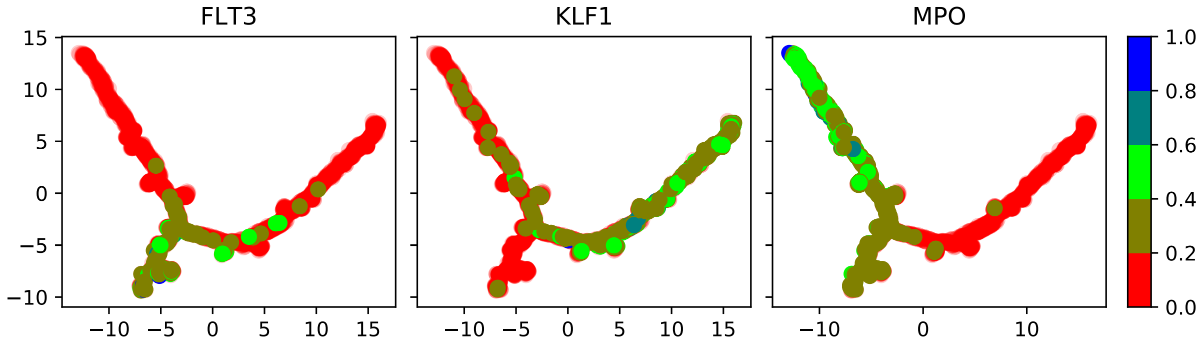

Figure 4: Hematopoiesis marker genes in Monocle latent space.

shown in Figure 5 (a). A significant portion of the spline branching times are near the end of pseudotime due to transitory gene expression which we discuss below. In Figures 5 (b)-(c) we show the branching times for ten marker genes that have been found to show significant evidence of branching. The colours reflect which branch is up-regulated after the most-likely branching time. For all GMP markers the same branch is upregulated (brown) whereas for the MEP markers the alternative branch is upregulated. Gene expression profiles are shown in Figure 6 for the GMP markers. The PRTN3 and CTSG markers are highly expressed and show clear branching behaviour; as a result they are the three top-ranked genes in terms of the log Bayes factor. In contrast, the CEBPA marker is lowly expressed and ranked 185th as the branches are less clearly separated. The profiles for some of the GMP markers are shown in Figure 7. When examining the individual gene expression for the APOE marker (Figures 7 (c)-(d)), the gene expression exhibits a transitory phase where the magenta branch is initially upregulated after the branching event but then is downregulated. This behaviour turns out to be quite common and can lead the spline-based approach to fail (Figure 7 (d)). This is because after the transitory phase is completed, it is likely the spline predictions for each branch will intersect again. In the spline approach the latest intersection point between the two fitted splines is selected as the branching point (**?**). This results in identifying the post-transitory phase point as the branching point. Transitory gene expression is also evident for other genes (Figure 8) wherein the spline approach similarly identifies the post-transitory phase as the dominant branching point.

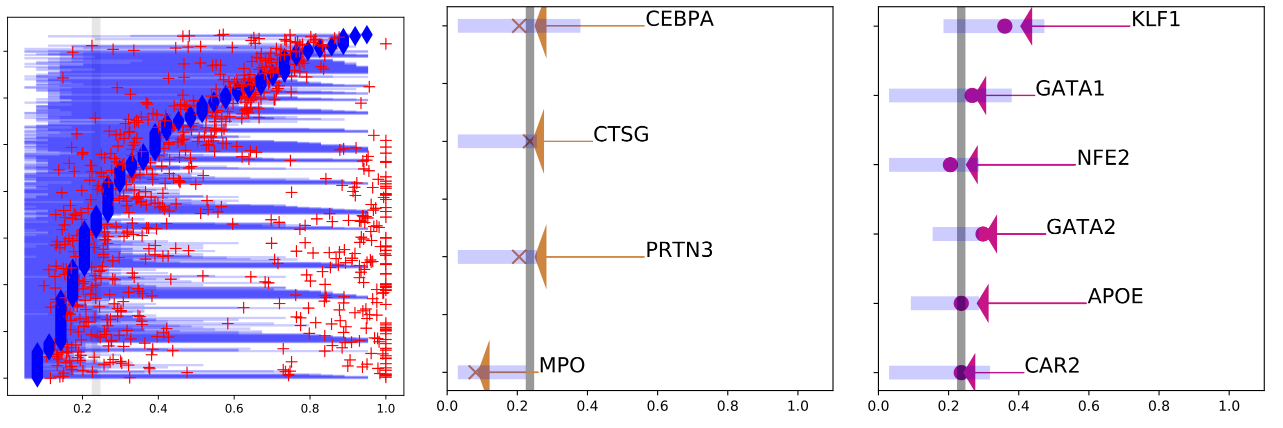

(a) Posterior branching times (b) Posterior branching for GMP marker genes (c) Posterior branching for MEP marker genes

Figure 5: Posterior summary of 737 genes identified as branching and selected marker genes for the hematopoiesis data. The genes are ordered by the branching location. The spline estimation is shown as red crosses and the global branching time by a vertical grey bar (b=0.21).

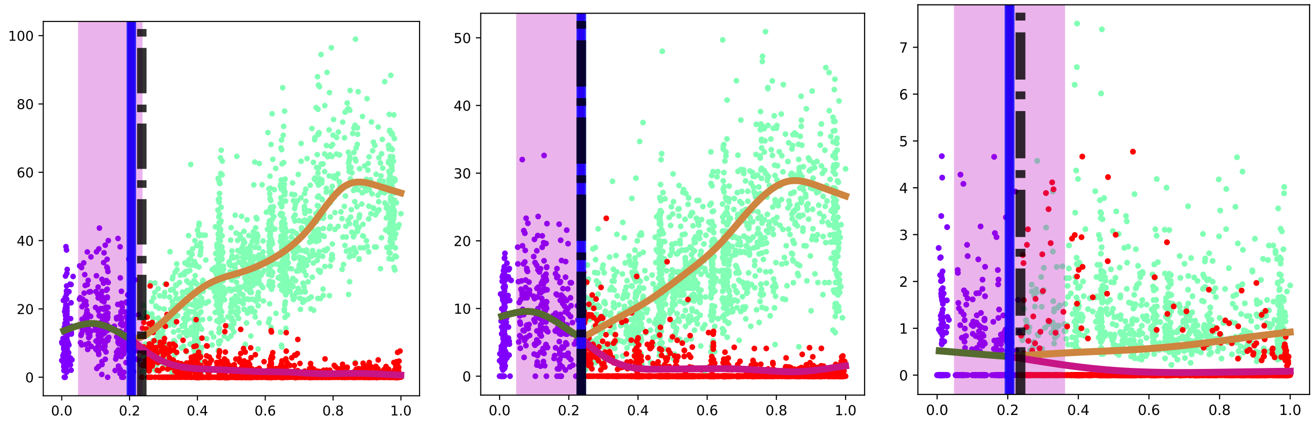

(a) PRTN3 (b) CTSG (c) CEBPA

Figure 6: Hematopoiesis gene expression: BGP estimation for GMP marker genes. The global branching time (dashed black vertical line), BGP branching point mode (blue vertical line) and 98% posterior intervals (magenta vertical span) are also shown. The cells are marked according to the global allocation estimated by the DDRTree algorithm.

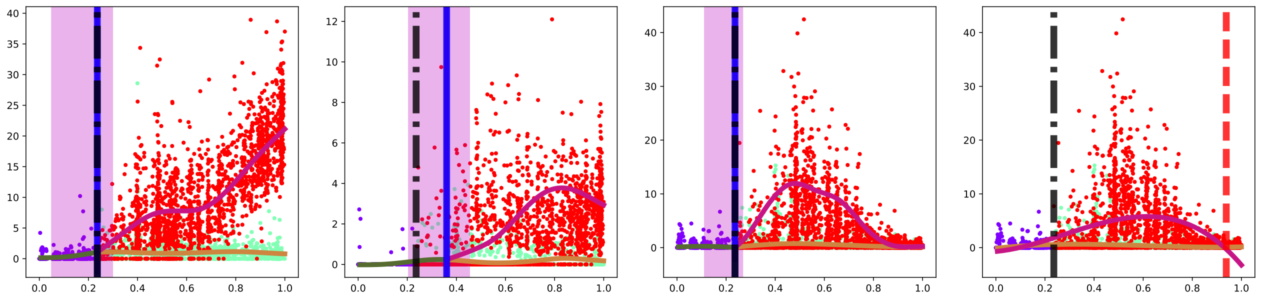

(a) CAR2 (b) KLF1 (c) APOE (d) APOE Spline

Figure 7: Hematopoiesis gene expression: BGP estimation for MEP marker genes. Also shown the spline fit for the APOE gene which exhibits transitory gene expression in one of the branches. The global branching time (dashed black vertical line), spline branching point mode (red vertical line) and 98% posterior intervals (magenta vertical span) are also shown. The cells are marked according to the global allocation estimated by the DDRTree algorithm.

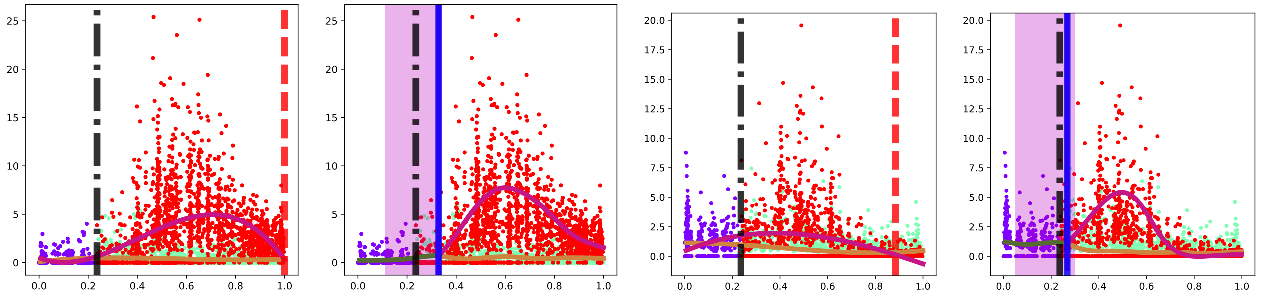

(a) VAMP5 Spline (b) VAMP5 BGP (c) ZYX Spline (d) ZYX BGP

Figure 8: Hematopoiesis gene expression: contrasting spline and BGP fits under transitory gene expression. The global branching time (dashed black vertical line), spline branching point mode (red vertical line) and 98% posterior intervals (magenta vertical span) are also shown. The cells are marked according to the global allocation estimated by DDRTree.
